# Impact of DAA Treatment on Cardiovascular Disease Risk in Chronic HCV Infection: An Update

**DOI:** 10.3389/fphar.2021.678546

**Published:** 2021-05-11

**Authors:** Hrvoje Roguljic, Vjera Nincevic, Kristina Bojanic, Lucija Kuna, Robert Smolic, Aleksandar Vcev, Dragan Primorac, Andrijana Vceva, George Y. Wu, Martina Smolic

**Affiliations:** ^1^ Faculty of Medicine Osijek, J. J. Strossmayer University of Osijek, Osijek, Croatia; ^2^ University Hospital Osijek, Osijek, Croatia; ^3^ Faculty of Dental Medicine and Health Osijek, J. J. Strossmayer University of Osijek, Osijek, Croatia; ^4^ Health Center Osijek, Osijek, Croatia; ^5^ St. Catherine Specialty Hospital, Zabok, Croatia; ^6^ Eberly College of Science, The Pennsylvania State University, State College, PA, United States; ^7^ The Henry C. Lee College of Criminal Justice and Forensic Sciences, University of New Haven, West Haven, CT, United States; ^8^ Medical School, University of Split, Split, Croatia; ^9^ Medical School, University of Rijeka, Rijeka, Croatia; ^10^ Medical School REGIOMED, Coburg, Germany; ^11^ Medical School, University of Mostar, Mostar, Bosnia and Herzegovina; ^12^ Department of Medicine, Division of Gastroenterology-Hepatology, University of Connecticut Health Center, Farmington, CT, United States

**Keywords:** hepatitis C virus, direct antiviral agents, cardiovascular disease, diabetes mellitus, dyslipidemia

## Abstract

Hepatitis C virus (HCV) infection is a systemic disease associated with multiple significant extrahepatic manifestations. Emerging studies indicate association between the HCV infection and a higher incidence of major adverse cardiovascular events such as: coronary artery disease, heart failure, stroke and peripheral artery disease, when compared to general population. Atherosclerosis is a common pathophysiologic mechanism of cardiovascular disease (CVD) development which is the leading cause of mortality in the Western world. Proposed mechanisms of HCV-induced atherosclerosis includes systemic inflammation due to the chronic infection with increased levels of pro-atherogenic cytokines and chemokines. Furthermore, it has been demonstrated that HCV exists and replicates within atheroschlerotic plaques, supporting the theory of direct pro-atherogenic effect of the virus. Direct acting antiviral agents (DAAs) represent a safe and highly effective treatment of HCV infection. Beside the improvement in liver-related outcomes, DAAs exhibit a beneficial effect on extra-hepatic manifestations of chronic HCV infection. Recently, it has been shown that patients with chronic HCV infection treated with DAA-based therapeutic regimes had a 43% reduction of CVD events incidence risk. Moreover, eradication of HCV with DAAs results in a significant positive effect on risk factors for cardiovascular disease, despite a general worsening of the lipid profile. This positive effects is mainly due to an improvement of endothelial function and glucose metabolism. Although DAA treatment is associated with a beneficial impact on cardiovascular events, further studies are needed to fully elucidate the mechanisms responsible.

## Introduction

About 70 million of people around the world are infected with hepatitis C virus (HCV) (Poller et al., 2018). HCV infection is one of the leading causes of chronic hepatitis and cirrhosis representing global health burden, even in the economically developed countries (Dash et al., 2020). Prevalence rates of chronic HCV infection are ranging between 1 and 5% in Europe and 1–4% in USA, depending on the geographic area, patients age and risk factors within population (Lee et al., 2019).

Most of HCV-infected individuals develop chronic infection characterized with an asymptomatic course. Along with the well-known long-term complications, such as liver cirrhosis and hepatocellular cancer, HCV infection is associated with numerous extrahepatic manifestations. Emerging evidence suggests that chronic HCV infection is a systemic disease characterized including various immune-related disorders, metabolic (glucose and lipid) alterations, neuropsychiatric diseases, kidney damage and cardiovascular disease (Nevola et al., 2021a). The chronicity of hepatitis C, as well as the presence of the virus in non-hepatic tissues creates a favorable milieu for the development of potential pathogenic impact on extrahepatic systems and organs (Mohanty et al., 2019).

During the past decade, direct-acting antiviral agents (DAAs) were introduced into the treatment of HCV infection as an effective and safe therapeutic option with fewer side effects in comparison with interferon-based therapies (Ioannou and Feld, 2019). Treatment with DAAs improves liver related outcomes such as liver cirrhosis and hepatocellular carcinoma. DAA-based regimens are characterized by >90% sustain viral response (SVR) which allows us to precisely evaluate effects of this group of medications on extrahepatic manifestations of chronic HCV infection (Fabrizi et al., 2019).

### Cardiovascular Manifestations of HCV Infection

**FIGURE 1:**
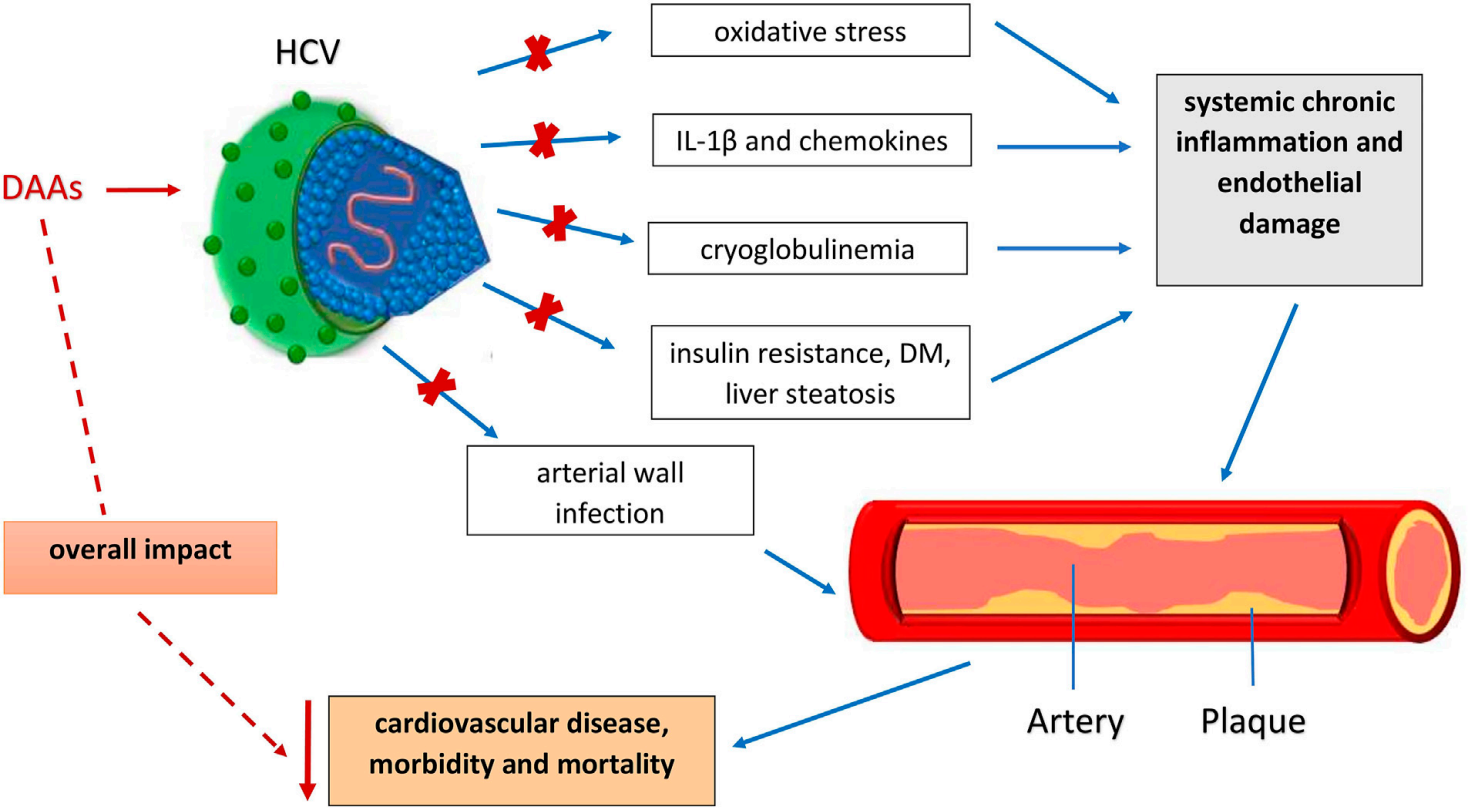
Influence of different factors responsible for development of atherosclerosis and CVD in HCV infected patients and HCV eradication by DAA drugs and positive effects on cardiovascular outcomes. DAA - Direct-acting antiviral agents; HCV - Hepatitis C virus; IL-1β - Interleukin-1β.

Over the last two decades, a large number of cohort studies, systemic reviews and meta-analyses have been conducted to elucidate an association between the HCV infection and the risk of the cardiovascular diseases (CVD). The latest evidence support the hypothesis of chronic HCV infection as an independent risk factor for subclinical and clinical CVD (Babiker et al., 2017; Iorga et al., 2020; Kuna et al., 2019; Kuan et al., 2019) and higher cardiovascular mortality (Domont and Cacoub, 2016; Poller et al., 2018). Among various CVDs, HCV mainly promotes atherosclerosis. Of the various proposed underlying pathophysiological mechanisms, the crucial one is that HCV infection-induced hepatic and systemic inflammation (Zampino et al., 2013) results in increased levels of pro-atherogenic chemokines and cytokines (interleukin (IL)-6, tumor necrosis factor (TNF)-α, C-reactive protein (CRP) and fibrinogen) and an imbalance between the anti- and pro-inflammatory cytokines (TNF-α/IL-10, IL6/IL-10) (Babiker et al., 2020; Zampino et al., 2013). In addition, a second pathophysiological mechanism is direct invasion of the arterial wall. This direct pro-atherogenic role of HCV has been shown to result in vascular inflammation (Mohanty et al., 2019). HCV has been shown to exist and replicates within atherosclerotic plaques causing arterial inflammation, likely by the pro-inflammatory cytokine interleukin-1β (Costantini et al., 2010). Furthermore, many direct and indirect immunological and biological mechanisms are included in HCV-triggered atherogenesis. These include oxidative stress, mixed cryoglobulinemia, endothelial dysfunction, insulin resistance (IR), type 2 diabetes, steatosis and other components of the metabolic syndrome as shown in Figure 1 (Adinolfi et al., 2014; Petta et al., 2018; Adinolfi et al., 2020a). HCV stimulates atherogenesis through the pro-inflammatory cytokine, interleukin-1ß causing arterial inflammation (Adinolfi et al., 2012; Adinolfi et al., 2018). In this regard, HCV proteins have a significant role in increasing oxidative stress and inducing chronic inflammation and endothelial damage (Iorga et al., 2020). HCV also interferes with glucose and lipid metabolism increasing factors that produce atherosclerosis such as IR, liver steatosis and diabetes (Iorga et al., 2020). Apart from common CVD risk factors, vasculitis represents a potential trigger of CVD events in HCV patients mostly through the development of cryoglobulinemia. HCV infection is responsible for more than 80% of mixed cryoglobulinemia cases as determined by the presence of cold-precipitating antibodies (Terrier et al., 2013; Ragab and Hussein, 2017).

The first study demonstrating the association of HCV infection with an increased risk of carotid atherosclerosis was published in 2002 (Ishizaka et al., 2002). Numerous studies that followed over the last 20 years demonstrated a definite and direct impact of HCV infection in the initiation of atherosclerotic plaques and increases in intima-media thickness. These were found to be independent of other risk factors, but predisposed individuals to the premature development of cardiovascular disorders (Adinolfi et al., 2012; Petta et al., 2012). To the contrary, several studies reported no association between HCV infection and atherosclerosis (Masiá et al., 2011). According to recent meta-analyses, it can be concluded that chronic HCV infection is associated with an increased risk of CVD and CVD-related mortality, carotid plaques and cerebrovascular events (Lee et al., 2019; Nevola et al., 2021b; Petta et al., 2016). Large artery, like carotid atherosclerosis and also small vessel disease could have a significant role in the pathogenesis of cerebrovascular accidents (CVA) among HCV-infected patients (Morgello et al., 2014). Regarding risk of CVA, many population cohort studies, reviews and meta-analyses over the last decade have consistently demonstrated the association between chronic HCV infection and a higher risk of stroke (Adinolfi et al., 2013; He et al., 2013; Ko et al., 2013; Ambrosino et al., 2016; Chew et al., 2017; Nevola et al., 2021a). In contrast to the above mentioned publications, only Younossi et al. reported contrary results and no significant association between HCV infection and CVA (adjusted OR: 0.58, 95% CI: 0.16–2.02). There are many confounding factors which may be responsible for the lack of consistency (Younossi et al., 2013). Published data also suggested that chronic HCV infection is a risk factor for the development of peripheral artery disease (PAD) independent of other risk factors (Butt et al., 2019). HCV-infected patients with concurrent comorbidity were found to have an even higher risk of developing PAD. Another study established that excess risk of developing PAD in HCV-infected patients was most pronounced in the first and third years of follow-up, and after that, it was negligible (Hsu et al., 2015). Except for atherosclerosis and PAD, the other most common cardiac manifestations were myocarditis, dilated or hypertrophic cardiomyopathies, coronary artery disease (CAD) and ischemic heart disease (Gill et al., 2016). Epidemiological data suggested that 17–37% of HCV-infected people also had a HCV-related heart disease (Matsumori, 2009.). It is assumed that both the direct viral cytotoxic and the indirect immune-mediated mechanisms of myocardial tissue damage are involved in the pathogenesis of HCV-induced cardiac disease (Tschöpe et al., 2020). The development of dilated cardiomyopathy in the chronic HCV infection has been connected to the genetic background of the patients, with HLA-DPB1*0901 and HLA-DRB1*1201 alleles being more prevalent in these patients (Sanchez and Bergasa, 2008). As a biomarker of cardiac dysfunction, higher levels of N-terminal pro-brain natriuretic peptide (NT-proBNP) were found in HCV-infected patients with impaired diastolic function (Che et al., 2012). However, the literature is not consistent regarding HCV associated cardiac disease. Most of the studies that found no connection to HCV infection involved myocardial infarction and ischeamic heart disease (Younossi et al., 2013). Regarding CAD, although various studies have shown mixed and ambiguous data, large meta-analysis (Ambrosino et al., 2016; Cacoub and Comarmond, 2019) determined a positive associations between HCV infection and CAD (Babiker et al., 2017).

### Effects of DAA Treatment on Cardiovascular Disease

Endothelial dysfunction has been predominantly recognized as the main change related to the pathogenesis of vascular diseases (Daiber et al., 2017). Petta et al., in a prospective study, examined the impact of a sustained viral response (SVR) on the levels of carotid atherosclerosis in patients with progressive liver fibrosis or cirrhosis. Collected data showed a decrease of the carotid intimal-medial thickening in patients on DAA regimens after (9–12 months) of follow up after viral eradication (Petta et al., 2018). Flow-mediated dilation (FMD), known as a CV risk marker, is a broadly recognized as a precise and non-invasive method of endothelial function evaluation (Di Minno et al., 2020). Di Minno et al. found that FMD altered from 4.52% at baseline to 9.39% after completing treatment with DAAs. Further, improvements in FMD have regularly been confirmed 12 weeks after the completion of treatment with DAAs. This evidence indicates a connection between viral eradication and endothelial function amelioration (Di Minno et al., 2020).

Several large multicenter studies confirmed an association of DAA-induced SVR with reduction in cardiovascular events as shown in Table 1(Cacoub and Saadoun, 2021). A recent retrospective study of 12,667 patients with chronic HCV infection treated with DAA demonstrated a 43% decrease in the risk of CVD events compared to the decrease of 22% in the risk of CVD events in patients using pegylated interferon and ribavirin regimen (Butt et al., 2019). However, the study had limitations due to retrospective nature, heterogeneity of the cohort which included mostly male patients (96,1%) and usage of ICD-9-CM and ICD-10 codes for end points. Adinolfi et al., in a recent prospective study which included 2,204 HCV patients, demonstrated that CV incidents (including acute coronary syndrome, stroke or TIA) after HCV clearance by DAA treatment were decreased by 2.0–3.5 fold, and the annual incidence of CV risk was highly decreased, by 0.68% (Adinolfi et al., 2020b). The effect is consistent irrespective of pre-treatment levels of liver fibrosis.

**TABLE 1:** Overview of the recent studies assessing the reduction in cardiovascular risk following DAA treatment.

	No. of patients (treated/untreated)	Follow up	Overall CVD outcomes	ACS or HF	CVA
Butt et al. (2019)	25,334 (12,667/12,667)	NA	HR= 0.57; 95% CI 0.51–0.65; *p* < 0.001	8.98 vs. 14.72; *p* < 0.001*	1.5 vs. 4.56; *p* < 0.001*
McGlynn et al. (2019)	49,332 (15,524/33,808)	DAA: 7207 patients/year	NA	aRR = 0.81 (0.30–2.20)	Ischemic: aRR=0.68 (0.42–1.10)
		Non-DAA: 64.823 patients/year			Hemorrhagic: aRR = 0.61 (0.22–1.70)
Adinolfi et al. (2020a)	2249 (1668/486)	28 months (median)	RR 0.379, *p* = 0.0002, 95% CI 0.221–0.628	NA	NA

ACS, acute coronary syndrome; CVA, cerebrovascular accidents; CVD, cardiovascular disease; HF, heart failure

* Incidence rates per 1000 patient/years.

Along with the potentially beneficial impact on CVD adverse events, DAA treatment is characterized by a favorable cardiovascular safety profile. DAAs treatment utilized in a national Egyptian study for HCV infection treatment in patients with and without liver cirrhosis, did not cause any change in QTc interval. According to the study reported by Biomy et al., the cardiovascular impact of DAAs was assessed in 170 patients with HCV, and no arrhythmias were noted (Biomy et al., 2017). A more recent study on DAAs in Egyptian patients also showed a benign on cardiovascular safety profile (Ibrahim et al., 2020). In addition, treatment with DAAs was not associated with higher rates of liver and kidney complications, or more frequent hospitalizations (McGlynn et al., 2019).

These data strongly suggest that HCV is an independent, non-traditional risk factor for CVD. Evidently DAA treatment and SVR achievement reduces cardiovascular risk (Ibrahim et al., 2020). In addition, eradication of HCV with DAA results in notable amelioration of endothelial function in patients with chronic hepatitis. It is most probable that DAA treatment will reduce the prevalence of cardiovascular events in patients with chronic hepatitis C (Adinolfi et al., 2020b), but further research is needed to evaluate the long-term effects of that.

### Changes in Metabolic Status of DAA Treated Patients

Beside the direct impact on CVD outcomes, eradication of HCV with DAA treatment shows beneficial effect on glucose homeostasis, a major risk factor for CVDs development. During clinical studies it was observed that HCV-infected individuals have a significantly higher prevalence of insulin resistance, as well as a higher risk for development of diabetes mellitus type II in comparison with uninfected subjects (Petta and Craxì, 2020). Achievement of virological eradication in DAA-treated patients without history of diabetes is associated with an improvement in glucose metabolism parameters and insulin homeostasis (Adinolfi et al., 2018; Gualerzi et al., 2018). Additionally, a decreased incidence of diabetes was observed in HCV patients treated with DAAs (Adinolfi et al., 2020). Further, diabetic patients during treatment with DAA regimens and after SVR exhibit lower levels of fasting glucose as well as hemoglobin A1c. Hence, a less intensive antidiabetic therapy requirements are needed in these individuals (Hum et al., 2017). The link between HCV eradication and glycemic control was further confirmed in liver transplant recipients with HCV and diagnosis of impaired fasting blood glucose, where SVR was associated with a reduction of fasting glucose serum levels (Saab et al., 2018).

Additionally, metabolic derangement is expected in chronic liver disease such as HCV-induced fatty liver due to the central role of hepatocytes in the lipid metabolism (Negro, 2014). In fact, HCV has notable impact on intracellular systems of the hepatocytes responsible for lipid metabolism. Uptake as well as secretion of HCV particles are dependent on apolipoproteins which results in significant changes in serum lipid profile. This viral hypolipidaemia is typically manifested by lower levels of LDL-C and total cholesterol in comparison with non-infected individuals while levels of HDL-C and triglycerides remain unaffected (Drazilova et al., 2018). Recent studies reported rapid changes in serum lipid levels during DAA based therapy manifested as an increase in LDL concentration and in total cholesterol, with no effect on HDL cholesterol levels (Hashimoto et al., 2016; Nevola et al., 2020). Further, during the 24 months follow-up period after the DAA treatment, a significant reduction of apoB serum values, a direct marker of LDL-C, was observed (Gitto et al., 2018). Interestingly, SVR achieved by DAA treatment regimens was associated with a significant reduction of triglyceride serum levels in comparison with IFN-based therapy (Carvalho et al., 2018). Taken together, eradication of HCV had an unfavorable effect on lipid profile consequently increasing the risk of cardiovascular disease development. Irrespective of this adverse effect on lipid profile, SVR was associated with improved overall cardiovascular mortality by eliminating numerous other detrimental effects of HCV.

## Discussion

Hepatitis C virus is no longer considered as an exclusively hepatotropic pathogen. Moreover, it is well known that chronic hepatitis C is a systemic disease. Current evidence strongly suggests that this commonly chronic infection affects a broad spectrum of extrahepatic organs causing multiple non-hepatic complications. Numerous studies have linked hepatitis C with higher incidences of CVD and also with an increased cardiovascular mortality (Sagnelli et al., 2020). Obviously, long term presence of the HCV along with a chronic inflammatory status, directly or indirectly, can be associated with the premature development of the diffuse atherosclerosis. Beside the pro-atherogenic effect, chronic hepatitis C is characterized with insulin resistance, renal impairment and cryoglobulinemia, conditions associated with increased risk for CVD. Although all mechanisms of atherogenicity of HCV have not been fully elucidated, chronic hepatitis C should be considered as an independent cardiovascular risk factor. In addition, Farrag and his colleagues have suggested that DAA antiviral regimens in HCV subjects may have a cardiotoxic effect as evidenced by global longitudinal strain. Although, this negative impact is more pronounced in patients with basic impairment of left ventricular function (Farrag et al., 2021). In addition, lipid metabolism has been reported to have been negatively impacted by the achievement of SVR by DAAs with a significant increase in LDL-C, a well-known major factor in the development of heart disease. Along this adverse effect on the lipid profile, the eradication of HCV is accompanied with an increased central arterial stiffness, a condition associated with deleterious vascular phenotypes in several diseases, such as atherosclerosis and renal disease (Chen et al., 2021). In addition, DAA-treated patients exhibit an increase in body weight due to the improvement of general metabolic status.

Nevertheless, the impact of the virus eradication with DAAs regimens has been shown to have an overall significant impact on reduction of cardiovascular adverse events confirming HCV as an independent CVD risk factor. Notable recent studies suggest that clearance of HCV by DAA treatment is associated with significant reduction of major cardiovascular events (Butt et al., 2019; Adinolfi et al., 2020). Further, achievement of SVR had beneficial impact on other extra-hepatic manifestations of chronic hepatitis C associated with adverse cardiovascular events. This reduction in the risk referred to all forms of major CV events and was not dependent of the stage of liver disease. However, the beneficial impact of DAA-based therapy on long-term CVD outcomes remains to be assessed because the majority of studies done so far have been short term. Nevertheless, these novel insights into the HCV infection indicate that the treatment of HCV infection should be undertaken not solely to eliminate the virus, but also to decrease morbidity and mortality from extrahepatic disease, especially cardiovascular.

The body of evidence indicates that because HCV infection affects so many metabolic functions, treatment and elimination of the virus and the accompanying inflammation has beneficial effects on the cardiovascular, and other systems. This is consistent with the findings of other virual infections such as that of HBV, and COVID-19. Accordinly, these benefits may be considered when assessing cost-benefit analyses for individual and public health treatment decisions.
